# Myelin Repair as a Novel Mechanism for Ketamine’s Sustained Antidepressant Effects

**DOI:** 10.2174/011570159X349856241213144902

**Published:** 2025-01-16

**Authors:** Sen Wang, Chaoli Huang, Mengyu Wang, Lingxiao Di, Cunming Liu, Kenji Hashimoto, Chun Yang

**Affiliations:** 1 Department of Anesthesiology, The First Affiliated Hospital of Nanjing Medical University, Nanjing, 210029, China;; 2 The First Clinical Medical College, Nanjing Medical University, Nanjing, 210029, China;; 3 Chiba University Center for Forensic Mental Health, Chiba, 260-8670, Japan

**Keywords:** Arketamine, enantiomer, esketamine, ketamine, depression, myelination, oligodendrocyte

## Abstract

Depression is a prevalent mental disorder, affecting approximately 300 million people worldwide. Despite decades of research into the underlying mechanisms of depression, a consensus remains elusive. Recent studies have implicated changes in oligodendrocytes and myelin in the pathogenesis of depression. Conventional antidepressants may alleviate symptoms within weeks of use, but approximately one-third of patients do not respond to them. Ketamine exhibits rapid and sustained antidepressant effects in treatment-resistant patients with depression. Given the association between reduced myelination and depression pathology, alterations in myelination may be a key mechanism underlying ketamine's prolonged antidepressant effects. However, the exact role of myelination in ketamine's sustained antidepressant effects remains unclear. In this review, we summarize the relationship between demyelination and depression and discuss the potential mechanisms by which ketamine may exert its antidepressant effects by repairing myelin damage, offering new insights into the role of myelination in antidepressant mechanisms.

## 1 INTRODUCTION

Depression, a globally pervasive mental illness, affects approximately 3.8% of the population, including 5% of adults (4% of men and 6% of women). The World Health Organization reports that approximately 300 million individuals worldwide are afflicted with major depressive disorder (MDD) [1]. MDD is characterized by a persistent, pervasive negative emotional state and a significant loss of interest and pleasure in daily activities. Other symptoms include sleep disturbances, changes in appetite, psychomotor retardation or agitation, concentration difficulties, self-denial, self-deprecation, and in severe cases, suicidal ideation, or behavior [2]. It significantly interferes with patients' daily functioning and social abilities, markedly reducing academic and occupational performance [3]. Therefore, finding effective treatments for depression carries substantial economic and social importance [4]. Unfortunately, many patients exhibit poor responses to first-line treatments like selective serotonin reuptake inhibitors (SSRIs) and endure significant side effects [5]. Additionally, SSRIs and other conventional antidepressants typically require a few weeks to manifest therapeutic effects and necessitate daily administration [6, 7].

The finding that ketamine, as an *N*-methyl-D-aspartate receptor (NMDAR) antagonist, exerts rapid and sustained antidepressant effects represents a significant breakthrough in depression treatment [8-10]. Originally derived from phencyclidine (PCP), ketamine was first introduced for human use in the 1960s, as a novel anesthetic compound [11, 12]. Ketamine demonstrates rapid and sustained antidepressant effects in treatment-resistant patients with MDD, potentially through NMDA and gamma-aminobutyric acid (GABA) receptor modulation. However, the antidepressant mechanisms of ketamine may involve additional pathways beyond NMDAR blockade, as other NMDAR antagonists did not produce ketamine-like robust antidepressant effects in MDD patients [13-16]. Recent studies suggest that alterations in myelin may be a key mechanism underlying ketamine's long-lasting antidepressant effects, given that reduced myelination and demyelination are linked to depression, and ketamine can durably enhance myelination [17]. Research has increasingly focused on the relationship between ketamine's antidepressant effects and myelination in recent years. Therefore, this review aims to elucidate the mechanisms by which ketamine exerts its rapid and sustained antidepressant effects through myelin repair.

## 2 MYLIN AND OLIGODENDROCYTES

Myelin is a membranous structure that encases the axons of the majority of neurons in both the central nervous system (CNS) and the peripheral nervous system (PNS) of vertebrates. It consists of the extended plasma membranes of oligodendrocytes in the CNS and Schwann cells in the PNS, which concentrically wrap around nerve axons [18, 19]. Functionally, myelin enhances the swift transmission of nerve signals and offers metabolic support to the axons it ensheathes [20]. Myelin does not completely cover nerve axons; the exposed regions between the myelin segments are known as nodes of Ranvier [21]. By increasing axonal resistance and reducing membrane capacitance, myelin enables action potentials to “jump” from one node of Ranvier to the next, a process known as saltatory conduction [22]. The low capacitance of myelin requires only minimal energy to depolarize the membrane between nodes, resulting in much faster action potential transmission compared to unmyelinated axons [20]. Beyond these crucial insulation and conduction roles, myelin performs several key functions within the nervous system. It mechanically isolates electrical signals from the surrounding microenvironment, thereby protecting axons. Additionally, oligodendrocytes provide metabolic support to neurons and to regulate ion and water homeostasis [23, 24]. Oligodendrocytes can produce lactate, which can subsequently be transferred to axons to generate metabolic energy in the form of ATP [25]. In the brain, lactate is shuttled by the most abundant lactate transporters such as MCT1, MCT2, and MCT4 [23, 25].

Oligodendrocytes are one of the primary types of glial cells in the CNS, alongside microglia and astrocytes. In addition to myelinating oligodendrocytes, the CNS contains two non-myelinating oligodendrocyte populations: mature oligodendrocyte precursor cells (OPCs) and perineuronal/satellite oligodendrocytes [26]. Oligodendrocytes originate from OPCs, which comprise only 5%-8% of glial cells and are uniformly distributed in both white matter and grey matter [27]. Before reaching their final maturation involving myelin formation, oligodendrocytes undergo many developmental stages. Characterizing these stages solely based on morphological criteria is often inadequate, both *in vivo* and *in vitro*. Therefore, the different states of oligodendrocytes are identified based on the expression of various developmental markers. Oligodendroglial cell lineage markers such as Olig1, Olig2, Sox10, and Nkx2.2 are expressed in all cells of the lineage, while OPCs and pre-oligodendrocytes are specifically characterized by the expression of platelet-derived growth factor receptor α (PDGFR-α) and neuron-glial antigen 2 (NG2) [28, 29]. Mature, differentiated oligodendrocytes are characterized by the production of myelin and several myelin-associated proteins. These myelin-associated proteins include myelin basic protein (MBP), which is expressed on the cytoplasmic surface of the plasma membrane, the transmembrane protein proteolipid protein (PLP), myelin-associated glycoprotein (MAG), the membrane marker galactocerebroside (GalC), and the surface marker myelin oligodendrocyte glycoprotein (MOG) [30-36].

In addition to promoting axonal myelination, oligodendrocytes play a crucial role in myelin regeneration. OPCs are the key cells in myelin regeneration, as they can generate new oligodendrocytes to replace old or damaged ones [37, 38]. It was previously thought that surviving oligodendrocytes did not participate in myelin regeneration. However, research by Duncan has shown that oligodendrocytes are associated with mature and remyelinated myelin, suggesting that surviving oligodendrocytes can indeed participate in myelin regeneration [39]. In the CNS, the most common causes of oligodendrocyte death are trauma, ischemia, and autoimmune attack. Oligodendrocyte death can lead to subsequent demyelinating lesions or result from primary myelin damage [40]. Although oligodendrocyte damage is not considered a primary cause of psychiatric disorders, oligodendrocytes are downstream targets in several psychiatric conditions, including MDD, schizophrenia, bipolar disorder, autism spectrum disorder, and attention deficit hyperactivity disorder (ADHD) [41, 42]. In chronic stress models, oligodendrocyte-specific genes are generally downregulated in the amygdala and prefrontal cortex (PFC). Dysregulations of oligodendrocytes and abnormalities in nodes of Ranvier are closely associated with depression [43].

## 3 RELATIONSHIP BETWEEN DEMYELINATION AND DEPRESSION

### 3.1 Depression

Depression is a common mental disorder with an unclear etiology. Research at various molecular levels suggests that the pathogenesis of MDD involves multiple complex and interrelated metabolic pathways [44]. Early studies indicated that monoamine oxidase inhibitors and tricyclic antidepressants could alleviate depressive symptoms by enhancing the activity of serotonin and norepinephrine [44, 45]. However, it is perplexing that the clinical onset of traditional antidepressants usually requires several weeks, while these drugs can almost instantly increase monoamine levels. Additionally, approximately one-third of patients with MDD do not respond to antidepressants that solely inhibit monoamine reuptake. Furthermore, restricting tryptophan, the precursor of serotonin, may not induce depressive symptoms in all patients [46, 47]. Therefore, the monoamine deficiency hypothesis may not universally apply to depressed patients, suggesting the involvement of other pathways and neurotransmitters in depression.

Stress is a major trigger for the onset and progression of mental disorders, including depression [48]. In mammals, the stress response is mediated by the hypothalamic-pituitary-adrenal (HPA) axis. The body terminates the stress response through negative feedback regulation by glucocorticoids secreted during stress, which activate glucocorticoid receptors (GRs) [49]. Elevated cortisol levels, overactivation of the HPA axis, and impaired negative feedback function have been observed in some patients with depression [50]. Furthermore, with age, HPA axis dysregulation becomes more prevalent in MDD.

Another hypothesis posits that the disruption of neurotrophic support is a key mechanism underlying the prominent symptoms and brain function changes in depression. Brain-derived neurotrophic factor (BDNF), a key member of the neurotrophic factor family, can activate tropomyosin receptor kinase B (TrkB) and P75 receptors. The BDNF-TrkB pathway plays a key role in depression [51, 52]. Alterations in BDNF expression levels are closely linked to cognitive impairment in depression [53]. There is a report showing alterations in BDNF and its precursor proBDNF in the postmortem brain and liver samples from MDD patients [54]. A substantial body of evidence indicates that BDNF levels are altered in patients with MDD, particularly showing reduced blood BDNF levels in acute MDD patients and in animal models of depression [55, 56]. The efficacy of traditional and rapid-acting antidepressants not only requires BDNF expression and its downstream signaling but also involves direct binding to the transmembrane domain of TrkB dimers, stabilizing the conformation of multiprotein complexes and promoting the binding of TrkB to BDNF [57, 58]. Through Trk receptors, neurotrophic factors can trigger intracellular signaling pathways, regulating cell fate, axonal growth, dendritic growth, and pruning, as well as overall neuronal function [59].

Recent studies also indicate that the gut-brain axis plays a crucial role in the pathogenesis of depression [60]. The gut microbiota is a key modulator of stress and inflammation, engaging in bidirectional communication with the endocrine and immune systems to regulate neuroinflammation, HPA axis activation, neurotransmitter transmission, and neuro-development [61-63].

### 3.2 Relationship between Demyelination and Depression

In the CNS, myelinating oligodendrocytes and the axons they ensheath form the white matter, a specialized structure integral to CNS function [64]. Recent neuroimaging studies have identified changes in brain structure and function among patients with MDD, including reductions in the volumes of cortical and subcortical structures [65, 66]. White matter pathways regulate unique abilities or behaviors. Studies involving isotopic and heterotopic transplantation into the adult rodent cortex have shown that white matter-derived OPCs differentiate into mature myelinating oligodendrocytes more effectively than genetically derived OPCs [67]. Alterations in neural traffic within certain white matter regions can initially result in complex behavioral dysfunctions, such as depression [64]. Diffusion tensor imaging (DTI) studies have shown that white-matter abnormality exists in individuals with MDD [68, 69]. Magnetic resonance imaging studies have revealed that early stages of depression are marked by a decrease in high signal intensity of white matter and abnormalities in myelin integrity, especially in the PFC, with these changes becoming progressively more severe as the disease advances. For example, significant reductions in white matter tract areas have been noted in the corpus callosum, cingulum, uncinate fasciculus, ventral PFC, left dorsolateral PFC, anterior limb of the internal capsule, left superior longitudinal fasciculus, parietal lobe, temporal cortex, and bilateral cortical or subcortical regions [70-79]. Patient samples diagnosed with depression exhibit reduced myelin content, axon numbers, and MBP expression, along with reactive gliosis in different brain regions [80, 81]. However, no significant change in the total number of OPCs within the white matter is observed [82].

Moreover, depression may also impact genes regulating myelination and myelin formation. Recent studies indicate that oligodendrogenesis and myelination in the PFC are highly responsive to stress experiences, including physiological or pathological stress responses [83, 84]. The PFC is a critical brain region involved in complex emotional and cognitive behaviors. Liu *et al*. [84] found that prolonged social isolation reduces the production of myelin genes and the formation of nuclear heterochromatin, leading to transcriptional and ultrastructural changes in oligodendrocytes in the PFC, ultimately impairing myelination. In stress-induced depressive mice, 69% of downregulated genes are related to myelination, such as MOG [85]. Microarray analysis of post-mortem tissues from patients with depression has shown significant downregulation of myelination or oligodendrocyte lineage-related transcriptional genes, particularly those crucial for myelin structure, such as 2′,3′-cyclic-nucleotide 3′-phosphodiesterase (CNP), myelin-associated glycoprotein (MAG), myelin and lymphocyte protein (MAL) [86], myelin oligodendrocyte MOG, myelin-associated oligodendrocytic basic protein (MOBP), peripheral myelin protein-22 (PMP22), and PLP, as depression severity increases [71, 87]. Consistent with these findings, research on animals exhibiting depression-like behaviors has demonstrated that exposure to chronic unpredictable mild stress (CUMS) in mice leads to alterations in approximately 30-40 genes, including oligodendrocyte-related genes like MBP, MOB, and CNP [65]. Our study also found changes in myelination during depression. Comparative transcriptomic analysis of the medial PFC (mPFC) and hippocampus in mice subjected to chronic social defeat stress (CSDS) revealed a significant decrease in the RNA expression levels of myelin protein-encoding genes in CSDS-susceptible mice compared to those either not exposed to CSDS or resistant to it. Notably, the myelin protein-encoding genes most affected included MBP, PLP, and MOBP [17]. Immunofluorescence staining techniques for detecting myelin in the mPFC and hippocampal regions of CSDS-susceptible mice showed morphological myelin damage, characterized by decreased axonal density, reduced myelin thickness and length, slower myelin growth, and significantly downregulated expression of MBP and PLP proteins. Additionally, western blot results from the brain tissue of patients with MDD indicated significantly reduced myelin protein expression, particularly MOBP, in the cerebral cortex [17]. These findings collectively indicate the presence of myelin damage in patients with depression.

## 4 ANTIDEPRESSANT EFFECTS OF KETAMINE

Berman *et al*. demonstrated that administering a single sub-anesthetic dose (0.5 mg/kg) of intravenous ketamine produced antidepressant effects in MDD patients within hours, lasting over 72 hours [88]. Despite the approval of (*S*)-ketamine nasal spray by the United States Food and Drug Administration (FDA) for use as an antidepressant, there are concerns about its long-term adverse effects [89]. Conversely, numerous preclinical studies indicate that (*R*)-ketamine has superior antidepressant effects compared to (*S*)-ketamine in rodent models [90].

**Fig. (1):**
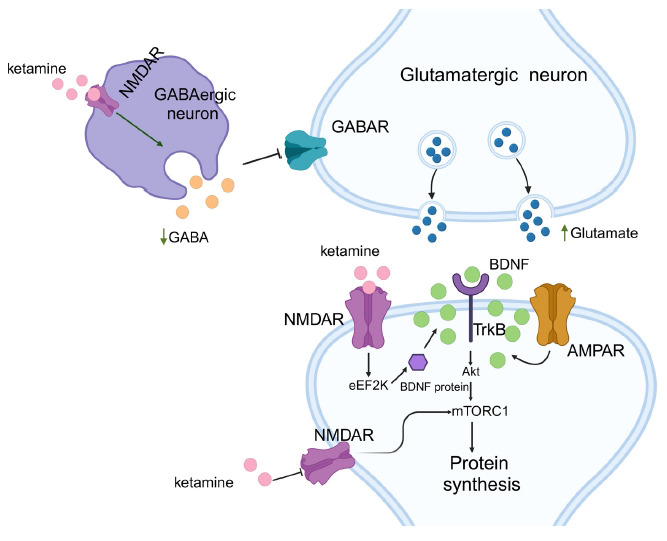
Signaling pathways underlying antidepressant effects of ketamine. Ketamine selectively blocks NMDA receptors expressed on GABAergic inhibitory interneurons that synapse on the dendrites, cell body and axon initial segment of pyramidal neurons. This leads to disinhibition of pyramidal neurons, increased firing and evoked glutamate release. The resulting glutamate surge stimulates postsynaptic AMPA receptors, leading to increased release of BDNF that activates TrkB and subsequent Akt/mTORC1 pathways. This ultimately leads to increased synthesis of proteins required for synaptogenesis. Ketamine also suppresses resting postsynaptic NMDA receptor activity, deactivating eEF2 kinase, resulting in reduced eEF2 phosphorylation, augmentation of BDNF synthesis and subsequent TrkB-mTORC1 activation. **Abbreviations**: AMPA, alpha-amino-3-hydroxy-5-methyl-4-isoxazolepropionic acid; BDNF, brain-derived neurotrophic factor; eEF2, eukaryotic elongation factor 2; GABAergic, gamma-aminobutyric acid-ergic; mTORC1, mammalian target of rapamycin complex 1; NMDA, *N*-methyl-D-aspartate; TrkB, tropomyosin receptor kinase B. (Image created with BioRender.com, with permission).

The mechanisms of ketamine's antidepressant action primarily include several aspects (Fig. **1**). By inhibiting NMDARs, ketamine reduces the activity of eukaryotic elongation factor 2 kinase (eEF2K), thereby rapidly increasing BDNF levels to produce antidepressant effects [91]. Ketamine can also block NMDARs on GABAergic interneurons, increasing glutamatergic neurotransmission and enhancing synaptic plasticity [92]. Additionally, alpha-amino-3-hydroxy-5-methyl-4-isoxazolepropionic acid receptor (AMPAR) antagonists can block ketamine's antidepressant effects in learned helplessness rats, suggesting that AMPARs also mediate ketamine's action [93]. BDNF has long been recognized as a critical mechanism in antidepressant efficacy, and ketamine is no exception. Within 30 minutes to 24 hours post-administration, ketamine significantly reduces immobility time in the forced swim test in control mice but not in BDNF knockout mice, indicating that BDNF is crucial for both rapid and sustained antidepressant effects of ketamine [91]. Ketamine can increase BDNF expression, activating the mammalian target of the rapamycin complex 1 (mTORC1) signaling pathway, resulting in increased postsynaptic density protein 95 (PSD-95), and exerting antidepressant effects [94]. A study using two enantiomers demonstrated that mTORC1 plays a role in the antidepressant effects of (*S*)-ketamine, but not (*R*)-ketamine, and that ERK plays a role in (*R*)-ketamine's antidepressant effects [95].

In behavioral despair models, combined use of ketamine and glycogen synthase kinase-3 (GSK-3) inhibitors rapidly activates the mTORC1 signaling pathway and increases excitatory postsynaptic currents in pyramidal neurons of the PFC, sustaining antidepressant effects for seven days. This suggests that ketamine's antidepressant mechanism involves reduced GSK-3 expression [96]. In contrast, a single administration of ketamine, but not GSK-3 inhibitor SB216763, produces a long-lasting antidepressant action in a chronic mild stress mouse model [97].

Ketamine is particularly effective in treating anhedonia in patients with depression, with brain imaging studies highlighting the potential role of the dopamine reward pathway in ketamine's antidepressant effects [98]. Preclinical studies in rodents show that ketamine stimulates dopamine neurons in the ventral tegmental area, leading to rapid and transient increases in extracellular dopamine levels in the mPFC [99]. Activation of dopamine D1 receptors increases the surface expression of NMDAR and AMPAR, promoting excitability and excitatory synaptic input of pyramidal neurons in the mPFC, potentially contributing to ketamine-induced synaptogenesis and enhancement [99]. In contrast, the dopamine D1 receptor antagonist SCH-23390 did not block the antidepressant effects of (*R*)-ketamine in CSDS-susceptible mice, suggesting that the dopamine D1 receptor may not play a major role in the antidepressant effects of (*R*)-ketamine [100].

Research also underscores the significance of the serotonin system in the antidepressant effects of ketamine. A single sub-anesthetic dose of ketamine quickly increases extracellular serotonin (5-HT) levels in the mPFC, and selective 5-HT_1A_ receptor agonists injected into the mPFC mimics ketamine's behavioral effects and stimulate mTORC1 signaling and synaptic protein expression in the mPFC [101]. Studies suggest that the lateral habenula (LHb) is involved in ketamine's action [102]. The LHb is a pathway connecting forebrain and midbrain monoaminergic nuclei. A group from Hailan Hu demonstrated that ketamine acts on NMDARs on LHb neurons, causing them to remain lodged in the ion channel, avoiding rapid metabolism internally. This results in sustained blockade of LHb neuron tonic firing, exerting long-lasting antidepressant effects [103].

Recent studies have also identified the involvement of the brain-gut-microbiota axis in depression, with (*R, S*)-ketamine and (*R*)*-*ketamine ameliorating depression phenotypes resulting from gut microbiota composition abnormalities [15, 104-107]. However, most current research on the cell type-specific mechanisms of ketamine's antidepressant effects has predominantly concentrated on the NMDA and AMPA receptors, with relatively few studies examining ketamine's effects on myelin and oligodendrocytes.

## 5 KETAMINE’S EFFECT ON DEPRESSION THROUGH REMYELINATION

### 5.1 Remyelination

Remyelination refers to the process of re-forming myelin after damage, specifically involving the re-differentiation of oligodendrocytes following their destruction. This regeneration is crucial for restoring axonal electrical signal conduction, supporting axonal metabolism, and preventing axonal degeneration, hence also termed myelin repair. In the CNS, the process of remyelination primarily includes the activation, migration, proliferation, and differentiation of OPCs [108]. This sequential process begins with the activation and recruitment of OPCs to the demyelinated areas, where they then differentiate into new oligodendrocytes, subsequently forming fresh myelin sheaths around the demyelinated axons. Upon demyelination, OPCs undergo morphological changes, extending pseudopodia to sense growth signals and migrate. Factors like platelet-derived growth factor (PDGF) and fibroblast growth factor (FGF) released at the injury site promote OPC proliferation and migration [109]. Additionally, the expression of various proteins, including transcription factors Nkx2.2 (NK2 homeobox 2), Olig2 (oligodendrocyte transcription factor 2), and SRY-box transcription factor 2 (SOX2), is upregulated [110]. Within days of demyelination, OPCs start migrating and dividing, a process requiring coordinated regulation by OPCs and other cells. The recruitment of OPCs to demyelinated areas largely depends on their migration and division, with immune system cells such as microglia and macrophages at the injury site being primary sources of activation and proliferation signals for OPCs. However, pathways such as Notch and WNT inhibit OPC differentiation, allowing sufficient proliferation before generating myelin to ensheath axons [110, 111].

After recruitment to demyelinated sites, OPCs undergo differentiation, extending around demyelinated axons to form dense myelin sheaths. In the final stage of oligodendrocyte differentiation, the thickness and length of newly formed myelin correlate with axonal diameter during development. However, in remyelination, new myelin sheaths are generally thinner and shorter than those formed during development, showing no significant linear relationship with the diameter of ensheathed axons [112, 113]. Additionally, other types of cells, including microglia, astrocytes, and immune cells, also play crucial roles in locating lesions, clearing myelin debris, and supporting regeneration during the remyelination process.

By staining for mature and immature OPC markers, we observed that the total number of cells within the oligodendrocyte lineage remained largely unchanged, indicating that OPC proliferation is not significantly affected by ketamine. However, analyzing the proportions of oligodendrocytes and OPCs within the lineage, we found increased OPC numbers and decreased mature oligodendrocytes in mice with depressed-like behaviors, suggesting an impediment in OPC differentiation into oligodendrocytes. This effect was reversed in ketamine-treated mice, implying that ketamine may promote OPC differentiation into mature oligodendrocytes in depression. Further, in wild-type mice treated with ketamine during peak OPC proliferation and differentiation phases, immunostaining for the proliferation marker bromodeoxyuridine (BrdU) and oligodendrocyte lineage markers revealed no significant change in BrdU-positive cell numbers during peak proliferation. However, during peak differentiation, the number and proportion of mature oligodendrocytes increased, while OPC numbers decreased, indicating that ketamine promotes OPC differentiation into oligodendrocytes rather than OPC proliferation in normal mice. Cell experiments corroborated these findings, suggesting that ketamine can repair impaired myelin by promoting OPC differentiation, yielding lasting antidepressant effects [17].

Moreover, in multiple sclerosis (MS), a disease primarily characterized by demyelination, ketamine has shown potential in promoting myelin repair. Researchers administered (*R*)*-*ketamine or saline to cuprizone-induced demyelination model mice for six weeks, and the subsequent MBP immunofluorescence staining of demyelinated brain regions revealed that (*R*)*-*ketamine significantly improved demyelination in the corpus callosum of cuprizone-treated mice [114]. Furthermore, (*R*)-ketamine improved the abnormal composition of gut microbiota and decreased levels of lactic acid in cuprizone-treated mice. There were significant correlations between demyelination in the brain and the relative abundance of several microbiomes, suggesting a link between gut microbiota and the brain. Moreover, (*R*)*-*ketamine facilitated remyelination in the brain after cuprizone withdrawal [114]. In the experimental autoimmune encephalomyelitis mouse model, (*R*)-ketamine also alleviated spinal cord demyelination [115]. Additionally, In the DTI studies, ketamine’s positive beneficial effects on white matter function in human MDD cases were also observed, suggesting a positive role of ketamine in myelination [116, 117]. These findings suggest that the beneficial effects of ketamine and (*R*)-ketamine may involve actions on myelin. Integrating current mainstream mechanisms of ketamine's antidepressant effects, we will explore its potential molecular mechanisms from perspectives of NMDARs, AMPARs, BDNF, and other glial cells.

### 5.2 Role of NMDARs and AMPARs in Myelination

Neurotransmitters are chemicals that transmit information between neurons or between neurons and effector cells. Glutamate is a crucial excitatory neurotransmitter in the CNS, participating in various physiological and pathological processes, including learning, memory, drug addiction, and psychiatric, and neurodegenerative diseases [118]. Glutamate receptors are classified into ionotropic (iGluR) and metabotropic (mGluR) receptors. Ionotropic glutamate receptors are further divided into three subtypes: NMDARs, AMPARs, and Kainate (KA) receptors [118, 119]. The AMPAR is a heterotetramer composed of four subunits (GluA1, GluA2, GluA3, and GluA4) [118]. The expression and activation of AMPARs commence early during development, with receptor density increase as OPCs mature. In immature OPCs, AMPARs and NMDARs are present at low densities, peaking in expression during later developmental stages. As OPCs differentiate into mature oligodendrocytes, the surface expression of AMPARs and NMDARs significantly decreases. Studies have shown that manipulating AMPAR expression in the oligodendrocyte lineage reveals the role of AMPAR activation and subunit composition in balancing OPC proliferation and mature oligodendrocyte survival.

NMDARs are complexes composed of at least seven subunits: GluN1, GluN2 (A-D), and GluN3 (A-B). NMDARs exhibit varying brain distributions and physiological characteristics based on their subunit composition [119, 120]. Functional NMDARs must include two NR1 subunits and two NR2 subunits, while NR3 subunits modulate NR1/NR2 functions and require binding with NR1 or NR2 to function. Unlike AMPARs, all NMDARs have high Ca^2+^ permeability [121]. The exact role and importance of NMDARs in OPCs in the CNS remain controversial. Some studies suggest that NMDARs do not play an active role in OPC maturation; OPCs lacking GluNR1 do not show significant changes in resting membrane properties or excitatory postsynaptic current (EPSC) numbers [122]. Additionally, no differences in OPC proliferation, morphology, or oligodendrocyte differentiation were observed [122, 123]. Consequently, some researchers conclude that NMDARs are not crucial for OPC development; instead, they support AMPAR function. For example, NMDAR knockout results in a 27% increase in Ca^2+^-permeable AMPARs. However, other studies indicate that while NMDARs may not affect OPC proliferation, they promote OPC migration. This effect has been confirmed *in vitro* and can be blocked by NMDA inhibitors and NMDAR subunit knockouts [124]. The expression of AMPARs begins early in development, and receptor density increases with the maturation of OPCs [125]. However, after OPC differentiation into mature oligodendrocytes, the density of AMPARs decreases approximately 12-fold, corresponding to a reduction in mRNA expression of all AMPA subunits. Research has indicated that manipulating AMPAR expression in the oligodendrocyte lineage identifies the role of AMPAR activation and subunit composition in balancing OPC proliferation and survival of mature oligodendrocytes [126]. These studies emphasize the significant role of AMPAR and NMDAR signaling in adult brain OPC proliferation and the generation of new oligodendrocytes and myelin.

Previous research has established that ketamine's antidepressant mechanisms are closely associated with the inhibition of NMDAR signaling and the activation of AMPA signaling. Thus, we hypothesize that ketamine promotes OPC differentiation into oligodendrocytes through these pathways. To test this hypothesis, we treated P7 and P14 mice with NMDAR inhibitors and AMPAR agonists. Results indicated that AMPAR agonists could mimic ketamine's effect in promoting OPC differentiation, while NMDAR inhibitors had a relatively weaker effect. This suggests that the AMPA signaling pathway may be the primary route through which ketamine promotes OPC differentiation. When cultured OPCs were treated with AMPAR inhibitors, ketamine's ability to promote OPC differentiation was significantly reduced. Furthermore, behavioral results showed that treating depressed mice with AMPAR inhibitors diminished ketamine's sustained antidepressant effects. In oligodendrocyte lineage staining, ketamine's promotion of OPC differentiation into oligodendrocytes was also inhibited [17].

### 5.3 Promoting Myelin Regeneration through Other Glial Cells

Microglia, as the resident macrophages of the CNS, play crucial roles in development, homeostasis, and response to injury [127]. Increasing evidence suggests that microglia not only support myelination but also promote axonal and myelin regeneration. Clusters of microglia are found in developing white matter and are believed to be involved in myelination [128, 129]. Early studies have shown that co-culturing microglia with oligodendrocytes stimulates the expression of myelin-specific proteins MBP and PLP in oligodendrocytes, indicating that microglia positively contribute to myelination [130]. Additionally, non-activated microglia enhance OPC survival and maturation by increasing PDGF-α signaling and regulating NF-kB activation [131]. Following myelin damage, microglia are among the first cells to be activated. Activated microglia can be classified into pro-inflammatory (M1) and immunomodulatory (M2) types based on cytokine expression [132]. One critical function of microglia or macrophages after demyelination is the removal of myelin debris, which inhibits OPC differentiation and thereby promotes myelin regeneration.

Our research discovered that the ubiquitin-related protein Uba52 was significantly elevated in CSDS-susceptible mice compared to control (no CSDS) and CSDS-resistant mice, and ketamine treatment reduced Uba52 levels. Since myelin degradation can be mediated by ubiquitin-dependent pathways, Uba52 may be associated with myelin damage in depression and ketamine's promotion of myelin repair. Additionally, Uba52 and its mediated ubiquitination activity are closely associated with microglial activity [133-135]. Therefore, we examined microglial activity in CSDS model mice. We observed that while the total number of microglia did not significantly change, the proportion of M2-type microglia increased significantly in CSDS-susceptible mice, with a trend of microglia shifting from M1 to M2 phenotypes in depression [17]. These phenomena indicate changes in microglial activity during the development of depression. However, ketamine treatment did not effectively alter the M1-to-M2 transition in microglia, suggesting that while microglial activity changes are related to myelin damage in depression, they are not closely associated with ketamine’s promotion of myelin repair [17].

These findings highlight the complex roles of glial cells, particularly microglia, in the context of myelin damage and repair in depression. While microglial activation and phenotype changes are linked to myelin damage, ketamine’s promotion of myelin repair seems to operate through different or additional mechanisms. Further research is necessary to clarify the precise mechanisms through which ketamine exerts its myelin repair and antidepressant effects.

### 5.4 Differential Effects of (*R*)-ketamine and (*S*)-ketamine on Myelination

Ketamine is a racemic mixture composed of equal amounts of two enantiomers: (*R*)-ketamine and (*S*)-ketamine. (*S*)-ketamine binds to NMDARs with four times the affinity of (*R*)-ketamine [136]. Additionally, (*S*)-ketamine has 3-4 times the anesthetic potency of (*R*)-ketamine but also has more adverse effects [137-139]. Despite the different affinities of (*R*)-ketamine and (*S*)-ketamine for NMDAR, (*R*)-ketamine exhibits more pronounced and prolonged antidepressant-like effects in animal models of depression [140, 141]. Based on these findings, we hypothesized that the more sustained antidepressant effects of (*R*)-ketamine might be related to myelination.

In our study, we treated CSDS-susceptible mice with (*R*)-ketamine and (*S*)-ketamine separately and found that (*R*)-ketamine was significantly more effective in repairing myelin damage associated with depressive-like phenotypes [17]. Moreover, (*R*)-ketamine better promoted OPC differentiation into mature oligodendrocytes compared to (*S*)-ketamine. Additionally, wild-type mice treated with (*R*)-ketamine for 14 days exhibited more efficient oligodendrocyte differentiation. When AMPAR inhibitors were used, the longer-lasting antidepressant effects of (*R*)-ketamine compared to (*S*)-ketamine were significantly reduced. These results indicate that (*R*)-ketamine is more effective in promoting myelin growth, contributing to its stronger antidepressant effects compared to (*S*)-ketamine. This may be due to (*R*)-ketamine’s superior ability to activate the AMPAR signaling pathway, while (*S*)-ketamine has a stronger antagonistic effect on NMDARs. This further supports the hypothesis that the AMPAR signaling pathway may be the primary molecular mechanism through which ketamine exerts its antidepressant effects by acting on myelin [17].

## 6 SUMMARY AND OUTLOOK

**Fig. (2):**
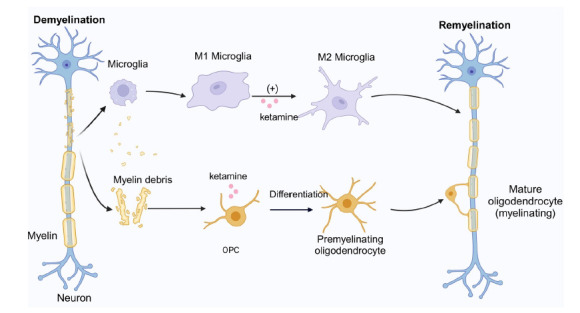
Possible mechanisms of ketamine’s effects on myelin repair. After myelin damage, the detached myelin debris activates microglia, which can remove the myelin debris that inhibits OPC differentiation. Ketamine can promote the transformation of microglia from M1 type to M2 type, thereby facilitating myelin repair. Additionally, ketamine can promote OPC differentiation into oligodendrocyte by acting on the AMPA receptors on the surface of OPCs, thereby promoting myelin repair. **Abbreviations**: AMPA, alpha-amino-3-hydroxy-5-methyl-4-isoxazolepropionic acid; OPC, oligodendrocyte precursor cell. (Image created with BioRender.com, with permission).

This review discusses the relationship between myelination and depression and explores the potential mechanisms through which ketamine exerts its long-lasting antidepressant effects *via* myelin (Fig. **2**). It emphasizes that ketamine's sustained antidepressant effects are likely attributed to its ability to activate AMPARs, which promote the differentiation of OPCs into mature oligodendrocytes and facilitates myelin repair. Given the current debate over the role of NMDARs in OPCs within the CNS and the fact that non-ketamine NMDAR inhibitors do not significantly block ketamine's promotion of OPC differentiation in mice with depression-like behaviors, it is suggested that ketamine is unlikely to exert its robust antidepressant effects on myelin *via* NMDARs.

Regarding the enantiomers of ketamine, (*R*)-ketamine has a stronger effect on promoting myelination and OPC differentiation compared to (*S*)-ketamine. This is likely due to (*R*)-ketamine's superior ability to activate the AMPAR signaling pathway. In summary, this review provides new insights into the long-lasting antidepressant effects of ketamine, suggesting that future antidepressant drug development could focus more on myelin-related targets. However, it remains unclear whether ketamine promotes myelination through other mechanisms and the role of Uba52 in this process. Further investigation is needed to elucidate these related actions and mechanisms.

(*S*)-ketamine is currently available for anesthesia and treatment-resistant depression, while clinical trials of (*R*)-ketamine are ongoing worldwide [16, 142]. Therefore, it is of great interest to compare the effects of these two enantiomers on depressive symptoms and demyelination in patients with MDD.

## LIST OF ABBREVIATIONS

ADHD: Attention Deficit Hyperactivity Disorder
BDNF: Brain-derived Neurotrophic Factor
CNS: Central Nervous System
CSDS: Chronic Social Defeat Stress
CUMS: Chronic Unpredictable Mild Stress
DTI: Diffusion Tensor Imaging
FGF: Fibroblast Growth Factor
GABA: Gamma-aminobutyric Acid
HPA: Hypothalamic-pituitary-adrenal
MBP: Myelin Basic Protein
MDD: Major Depressive Disorder
MS: Multiple Sclerosis
NMDAR: *N*-methyl-D-aspartate Receptor
OPCs: Oligodendrocyte Precursor Cells
PDGFR-α: Platelet-derived Growth Factor Receptor α
PNS: Peripheral Nervous System
